# Metastatic pediatric alveolar soft part sarcoma: a rare case report highlighting multidisciplinary treatment, molecular diagnostics, and novel therapeutic approaches

**DOI:** 10.3389/fped.2025.1638152

**Published:** 2025-08-14

**Authors:** Şule Çalışkan Kamış, Begül Yağcı

**Affiliations:** Department of Pediatric Hematology and Oncology, Adana Faculty of Medicine, Adana City Education and Research Hospital, University of Health Sciences, Adana, Türkiye

**Keywords:** alveolar soft part sarcoma, pediatric sarcoma, ASPSCR1-TFE3 fusion, targeted therapy, immunotherapy, molecular diagnostics, pediatric oncology, case report

## Abstract

**Background:**

Alveolar soft part sarcoma (ASPS) is an extremely rare soft tissue sarcoma, accounting for less than 1% of all soft tissue sarcomas. Approximately 5%–10% of ASPS cases occur in children and adolescents. Despite its indolent local course, its high metastatic potential necessitates comprehensive, multidisciplinary management.

**Case presentation:**

We present a pediatric patient diagnosed with metastatic ASPS originating from the left thigh. Initial management included wide local excision and adjuvant radiotherapy. Immunohistochemistry demonstrated strong nuclear TFE3 positivity, supporting the diagnosis. Although FISH analysis for ASPSCR1–TFE3 fusion was planned, technical limitations prevented archival imaging. Despite initial local control, the patient developed pulmonary, hepatic, brain, and intraabdominal metastases during follow-up. Serial imaging, including ^18^F-FDG PET/CT and brain MRI, revealed progressive disease with increasing metastatic burden in the lungs, mediastinum, brain, and distal colon. The patient underwent multiple systemic treatments over approximately 30 months, including tyrosine kinase inhibitors, an mTOR inhibitor (sirolimus), and immune checkpoint inhibition (pembrolizumab), alongside multiple courses of radiotherapy. The entire treatment timeline illustrates the complex, multidisciplinary management required for this ultra-rare malignancy.

**Conclusion:**

This case highlights the critical role of histopathological and molecular confirmation, multidisciplinary care, and emerging targeted and immunotherapy approaches in pediatric ASPS. Collaborative multicenter trials are urgently needed to establish evidence-based treatment strategies for this challenging disease.

## Introduction

Alveolar soft part sarcoma (ASPS) is an extremely rare mesenchymal soft tissue tumor first described by Christopherson et al. in 1952. It accounts for less than 1% of all soft tissue sarcomas and is characterized by the distinctive unbalanced translocation t(X;17)(p11;q25), which generates the ASPSCR1-TFE3 fusion gene (1, 2). This fusion protein acts as an aberrant transcription factor, upregulating the MET proto-oncogene receptor tyrosine kinase (MET) signaling pathway, which promotes tumor proliferation, angiogenesis, and early metastasis, making this axis a promising therapeutic target (2–4).

ASPS most commonly affects adolescents and young adults, with only about 5%–10% of cases occurring in children under the age of 15 (1, 5, 6). Tumors typically arise from the deep soft tissues of the lower extremities, especially the thigh and buttock region, but may also develop in other anatomical sites including the trunk, head and neck region, tongue, and orbit, particularly in younger patients (1, 2).

Clinically, ASPS is notorious for its insidious onset and slow local growth, which often results in delayed diagnosis (1, 5). Despite its indolent behavior, it has a strong propensity for early hematogenous metastasis, especially to the lungs and brain, and less commonly to bones and liver (1, 5). At diagnosis, a significant proportion of pediatric patients already present with metastatic disease, predominantly involving the lungs (1, 5). The tumor's pronounced vascularity can be readily identified on imaging, often demonstrating vivid contrast enhancement on MRI and CT scans (1).

Conventional chemotherapeutic regimens—including ifosfamide and doxorubicin, which are effective in other pediatric soft tissue sarcomas—generally show limited benefit in ASPS (1, 2). This intrinsic chemoresistance contributes to the poor prognosis in advanced cases and highlights the need for novel, molecularly guided strategies (2). In recent years, targeted therapies such as anti-angiogenic tyrosine kinase inhibitors (TKIs) including pazopanib, sunitinib, and cediranib, as well as mTOR inhibitors like sirolimus, have shown promise in achieving disease stabilization (2, 4, 5). Moreover, emerging evidence suggests that ASPS may express PD-L1, providing a rationale for the use of immune checkpoint inhibitors such as pembrolizumab and nivolumab, especially for refractory or progressive cases (3, 7, 8). However, robust pediatric-specific data for these novel therapies remain limited (4, 7, 9).

Given the rarity of ASPS in children and the absence of standardized treatment guidelines, each case presents unique diagnostic and therapeutic challenges that necessitate a comprehensive, multidisciplinary approach (1, 9). Herein, we report a pediatric patient with widely metastatic ASPS who was managed with multiple treatment modalities, including surgery, radiotherapy, targeted therapy, and immunotherapy, underscoring the importance of molecular confirmation and the ongoing need for innovative, evidence-based strategies for this ultra-rare malignancy (6, 8).

## Case presentation

A previously healthy 4-year-old girl presented with a painless swelling over the medial aspect of her left thigh that had been gradually increasing in size over several weeks. Magnetic resonance imaging (MRI) demonstrated a heterogeneous 4.5 × 5 cm soft tissue mass located in the mid-thigh, showing vivid contrast enhancement and high vascularity, features typical of alveolar soft part sarcoma (ASPS). Whole-body PET/CT revealed increased uptake in the primary mass with a SUVmax of 2.46 and multiple bilateral pulmonary nodules consistent with hematogenous metastases. This radiological pattern is consistent with the typically moderate FDG uptake reported for ASPS in the literature.

**Figure 1:**
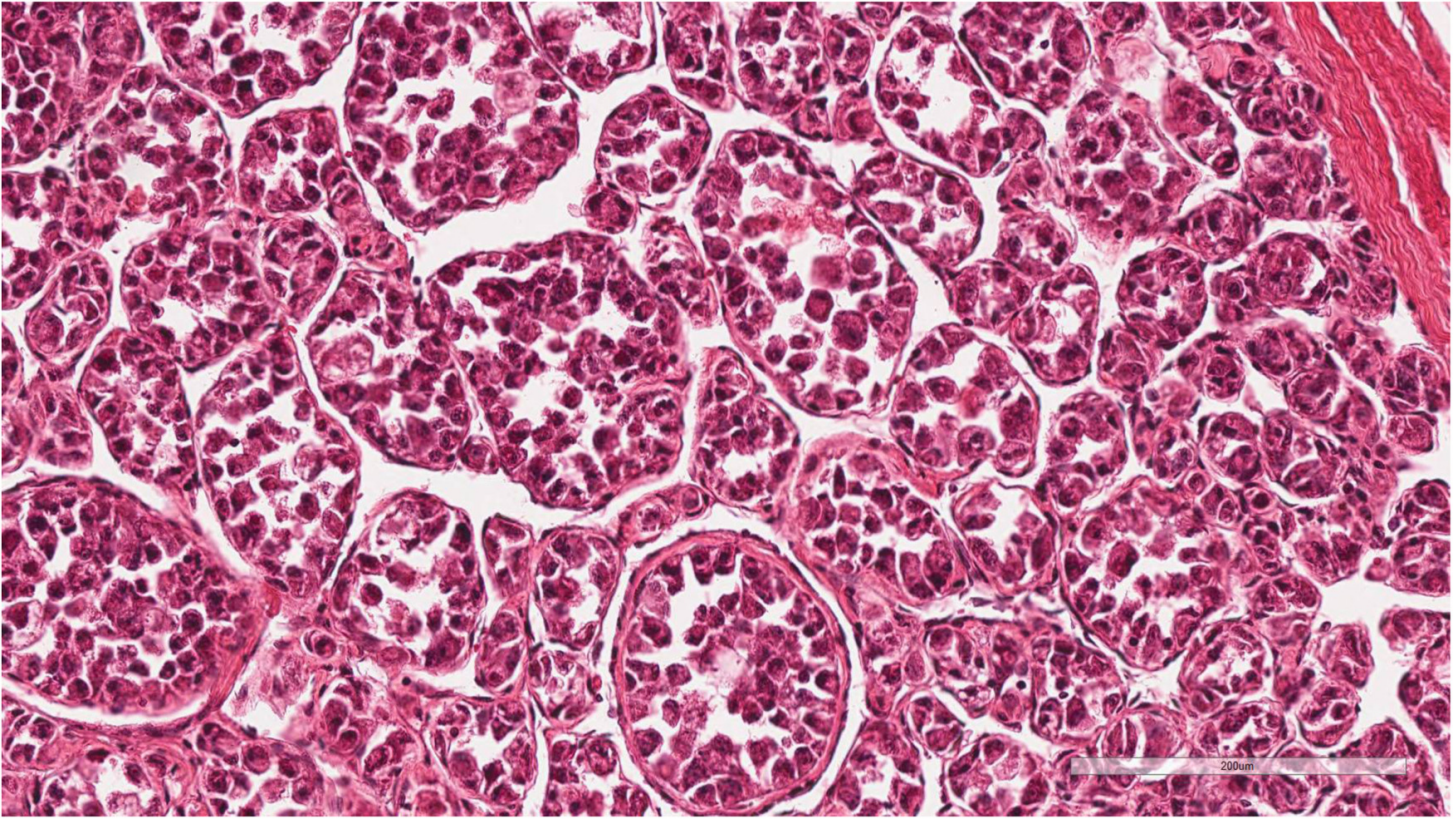
Histopathological findings of the pediatric patient diagnosed with alveolar soft part sarcoma (ASPS). The tumor shows large polygonal cells with abundant eosinophilic granular cytoplasm arranged in a pseudoalveolar pattern (Hematoxylin and eosin stain, 200×). Immunohistochemical staining demonstrated strong and diffuse nuclear TFE3 positivity as reported. Although fluorescence *in situ* hybridization (FISH) analysis for the *ASPSCR1-TFE3* fusion gene was planned, an archival image could not be obtained due to technical limitations. The diagnosis was further supported by multiple consistent pathology reports from different institutions.

Histopathological examination of the incisional biopsy specimen showed large polygonal tumor cells arranged in organoid nests with abundant granular eosinophilic cytoplasm forming a pseudoalveolar pattern (Figure 1). Immunohistochemical staining demonstrated strong and diffuse nuclear TFE3 positivity, supporting the diagnosis of alveolar soft part sarcoma (ASPS). Although fluorescence *in situ* hybridization (FISH) analysis for the *ASPSCR1-TFE3*fusion gene was planned, an archival image could not be obtained due to technical limitations. Multiple pathology reports from different institutions consistently confirmed the diagnosis of ASPS with reproducible histomorphological and immunohistochemical findings. No evidence of other primary tumor sites was identified on imaging.

The patient's initial management consisted of two cycles of ifosfamide-etoposide and two additional cycles of ifosfamide alone, administered according to the ARST0332 protocol for pediatric rhabdomyosarcoma due to the lack of standardized protocols for pediatric ASPS. However, only minimal response was observed. Complete surgical excision was then performed, achieving negative margins. Postoperative pathology revealed lymphovascular invasion and focal desmin positivity. Due to wound healing complications, adjuvant radiotherapy to the surgical site (45 Gy in 25 fractions) was delayed until week 10 post-surgery.

Subsequent systemic therapy was switched to the VAC-A regimen (vincristine, actinomycin D, cyclophosphamide, doxorubicin) to address residual pulmonary metastases. Restaging scans, however, demonstrated progression of lung lesions. Doxorubicin was discontinued after reaching the cumulative lifetime maximum dose. Given ongoing progression, multiple salvage regimens were administered sequentially: ICE (ifosfamide, carboplatin, etoposide) combined with the tyrosine kinase inhibitor pazopanib, followed by TCV (topotecan, cyclophosphamide, vincristine), and then VIT (vincristine, irinotecan, temozolomide). Only transient and partial responses were observed, with progression recurring on each regimen. The chronological sequence of systemic treatments, including doses, durations, and adverse events, is provided in Table 1.

**Table 1:** Chronological summary of systemic treatments, doses, durations, and adverse events.

Treatment line	Modality/drug	Dose & schedule	Duration	Adverse events
1st line	Ifosfamide-etoposide	Standard ARST0332	2 cycles	Neutropenia
2nd line	Wide local excision	–	–	–
3rd line	Radiotherapy (thigh)	45 Gy in 25 fractions	–	Grade 1 skin dermatitis
4th line	VAC-A regimen	Standard pediatric doses	4 cycles	Myelosuppression
5th line	ICE + Pazopanib	Ifosfamide, carboplatin, etoposide + 800 mg daily	6 months	Elevated liver transaminases
6th line	TCV regimen	Standard pediatric doses	4 months	Nausea, myelosuppression
7th line	VIT regimen	Standard pediatric doses	4 months	Diarrhea, neutropenia
8th line	Gemcitabine-docetaxel	Standard doses	3 months	Fatigue
9th line	Sirolimus	2 mg daily	12 months	Hyperlipidemia
10th line	Cranial radiotherapy	25 Gy	–	Headache, fatigue
11th line	Pembrolizumab	200 mg every 3 weeks	∼3 months (discontinued due to drug unavailability)	Mild fatigue, no immune-related AEs
12th line	Abdominal radiotherapy	25 Gy in 5 fractions	1 week	Mild GI discomfort

Gy, Gray; RT, radiotherapy; AEs, adverse events; Q3W, every 3 weeks.

Despite multiple systemic regimens, disease progression persisted. Following transient stability with gemcitabine–docetaxel, new hepatic metastases prompted the initiation of sirolimus, an mTOR inhibitor, which achieved stabilization of lung lesions for approximately 12 months. Eighteen months into sirolimus therapy, the patient developed new neurological symptoms, including headache and focal weakness. Brain MRI revealed a large left frontal lobe mass with surrounding vasogenic edema, confirmed by biopsy to be an ASPS metastasis. Focal cranial radiotherapy (25 Gy) was administered to the brain lesion, and pazopanib was reintroduced.

**Figure 2:**
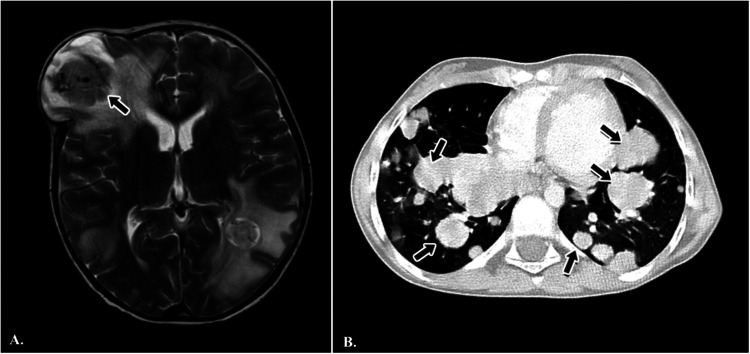
Radiological findings in a pediatric patient with metastatic alveolar soft part sarcoma (ASPS). **(A)** Axial T2-weighted brain MRI demonstrating a large metastatic mass in the left frontal lobe with surrounding edema (black arrow). **(B)** Axial chest CT scan showing multiple, bilateral, well-circumscribed pulmonary metastases of varying sizes (black arrows).

**Figure 3:**
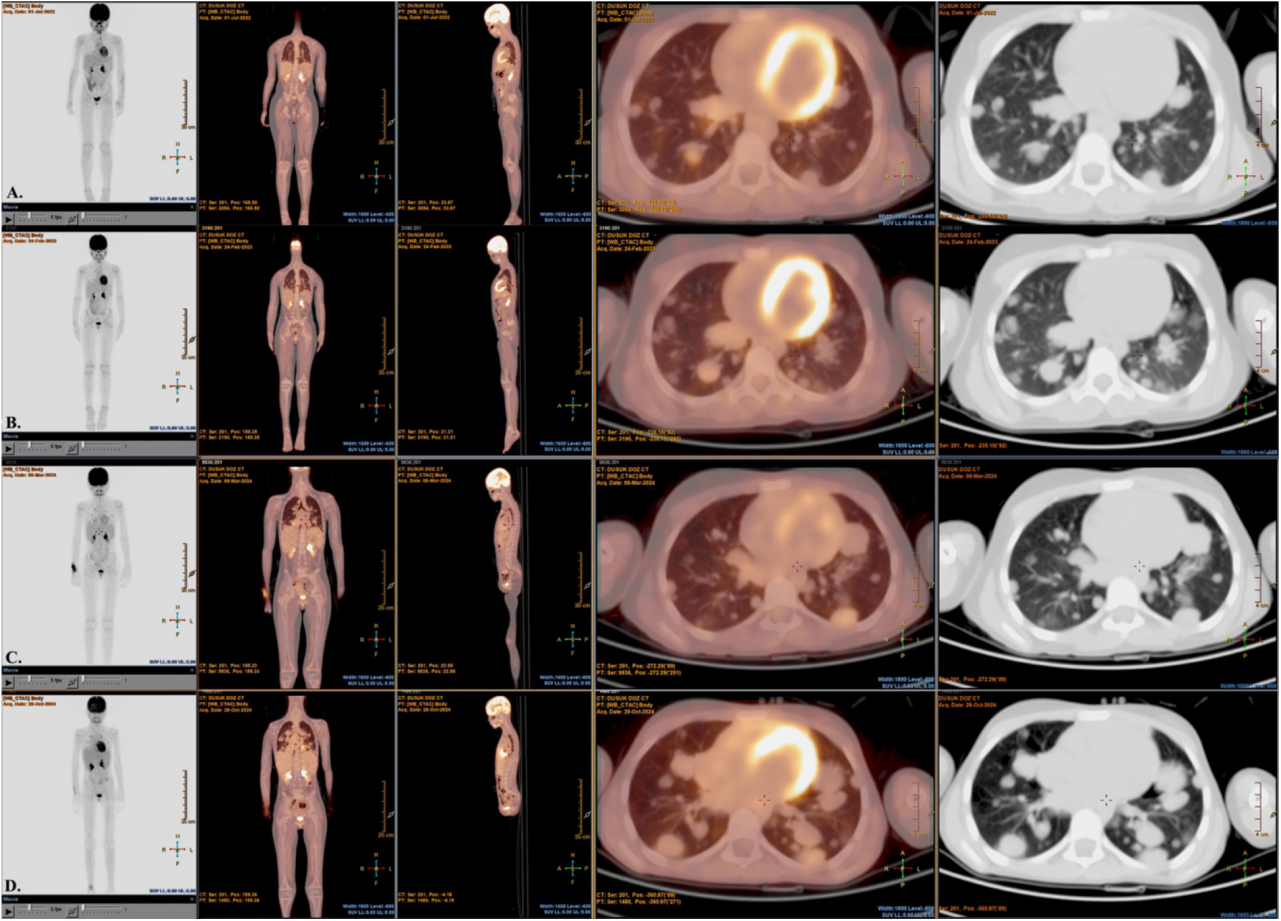
Serial ^18^F-FDG PET/CT images and nuclear medicine report summaries in a pediatric patient with metastatic alveolar soft part sarcoma (ASPS). **(A)** On 01 July 2022, PET/CT shows early bilateral pulmonary nodules with low-grade FDG uptake, consistent with initial metastatic involvement. **(B)** On 24 February 2023, there are multiple bilateral lung parenchymal nodules with mild–moderate FDG avidity, indicating interval progression from baseline. **(C)** On 08 March 2024, there is a further increase in both the number and FDG uptake of pulmonary metastases, with new FDG-avid mediastinal lymphadenopathy. **(D)** On 28 October 2024, follow-up demonstrates continued disease progression despite multimodal therapy, with extensive FDG-avid pulmonary and mediastinal lesions. FDG, fluorodeoxyglucose; PET/CT, positron emission tomography/computed tomography; ASPS, alveolar soft part sarcoma.

**Figure 4:**
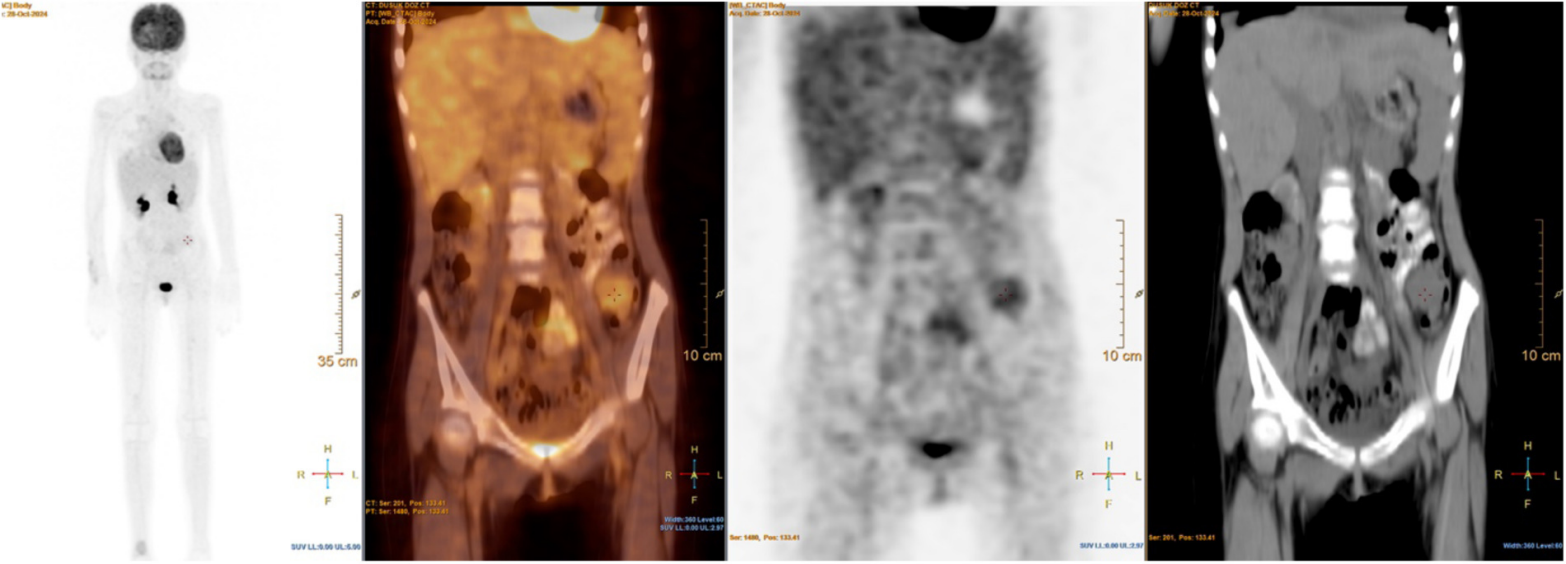
^18^F-FDG PET/CT images showing abdominal metastases in a pediatric patient with metastatic alveolar soft part sarcoma (ASPS). This figure demonstrates a newly detected minimally FDG-avid lesion in the distal colon, consistent with intraabdominal metastatic spread. This finding highlights the aggressive and widespread nature of disease progression despite intensive multimodal therapy.

Radiological reassessment demonstrated multiple well-circumscribed pulmonary metastases on thoracic CT and a large left frontal lobe metastasis on brain MRI with surrounding edema (Figures 2A,B). Serial ^18^F-FDG PET/CT scans performed on 01 July 2022, 24 February 2023, 08 March 2024, and 28 October 2024 revealed a gradual increase in both the number and metabolic activity of pulmonary and mediastinal metastases over time (Figure 3). In addition, PET/CT identified a newly emerging minimally FDG-avid lesion in the distal colon, consistent with intraabdominal metastatic spread (Figure 4). Despite ongoing intensive multimodal therapy, new metastatic foci continued to appear in the mediastinum, colon, and additional pulmonary sites, highlighting the aggressive and refractory course of the disease.

**Figure 5:**
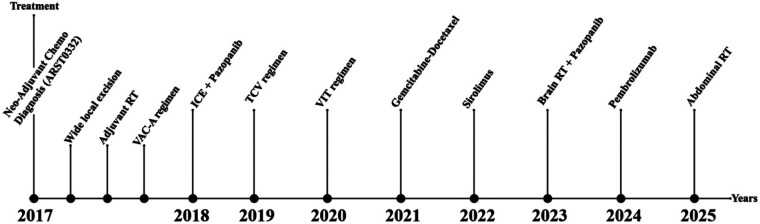
Chronological treatment timeline (2017–2025). This figure summarizes the entire chronological sequence of local and systemic treatments administered from the initial diagnosis at age four to the last follow-up. It illustrates the complex, multidisciplinary management of pediatric metastatic alveolar soft part sarcoma, including neoadjuvant chemotherapy, surgery, adjuvant radiotherapy, multiple systemic regimens, targeted therapies (mTOR inhibitors, TKIs), immune checkpoint blockade, and the recently planned palliative abdominal radiotherapy.

Based on multidisciplinary tumor board consensus, immune checkpoint inhibition with pembrolizumab was initiated but had to be discontinued due to drug access limitations. Palliative abdominal radiotherapy was subsequently planned to address intraabdominal metastases as part of supportive care. The overall treatment timeline from initial diagnosis to last follow-up is summarized in Figure 5.

In addition to systemic therapies, the patient also underwent multiple courses of palliative radiotherapy as summarized below. Over the course of the disease, the patient received palliative radiotherapy to control symptomatic metastases at various anatomical sites. In October 2023, whole-cranium radiotherapy was administered with a total dose of 25 Gy in 5 fractions using tomotherapy (IMRT), targeting diffuse metastatic involvement including the right frontal convexity. In January 2024, stereotactic radiotherapy was applied to a left frontal lobe brain metastasis at a dose of 18 Gy in a single fraction (80% isodose), utilizing a 7-field IMRT plan. In September 2024, radiotherapy was delivered to bulky right hilar and paracardiac thoracic lesions at a total dose of 25 Gy in 5 fractions with a 7-field palliative IMRT plan. Finally, in May 2025, abdominal radiotherapy was administered to a midline abdominal mass with a total dose of 25 Gy in 5 fractions, again using palliative IMRT. All radiotherapy interventions were planned in accordance with multidisciplinary tumor board recommendations, aiming to relieve symptoms, reduce tumor burden, and maintain quality of life.

## Discussion

Alveolar soft part sarcoma (ASPS) poses significant diagnostic and therapeutic challenges, particularly in pediatric patients, due to its rarity and indolent yet paradoxically aggressive clinical course (1, 5). The initial slow growth and non-specific presentation often result in delayed diagnosis, and unfamiliarity among clinicians can further complicate early recognition (1, 5). In our patient, as commonly reported in the literature, pulmonary metastases were present at diagnosis, with subsequent spread to the brain and liver, underscoring the tumor's strong predilection for early hematogenous dissemination (1, 2, 5).

Histologically, ASPS is characterized by large polygonal tumor cells arranged in nests or alveolar patterns with abundant eosinophilic cytoplasm and periodic acid–Schiff (PAS)-positive, diastase-resistant intracytoplasmic crystals (2). Strong nuclear TFE3 immunoreactivity, resulting from the ASPSCR1-TFE3 gene fusion, serves as a diagnostic hallmark and represents a critical molecular feature distinguishing ASPS from histologic mimics (2). This gene fusion activates multiple downstream pathways, including MET signaling, highlighting actionable therapeutic targets that have become the focus of emerging treatment strategies (2, 3).

Conventional cytotoxic chemotherapies such as doxorubicin, ifosfamide, vincristine, and etoposide have shown minimal activity in ASPS, with response rates remaining disappointingly low in both adult and pediatric series (1, 5). In our case, standard chemotherapy regimens, including ARST0332-based combinations and multi-agent salvage protocols, failed to achieve durable disease control, consistent with prior reports of intrinsic chemoresistance in ASPS (1, 2). For localized disease, complete surgical resection remains the mainstay of treatment and is associated with improved outcomes; however, in cases of widespread metastases, complete resection is seldom feasible (1). Radiotherapy may provide local control or palliation, especially for symptomatic sites such as brain metastases, as was performed for our patient's frontal lobe lesion (5).

Over the past decade, there has been a paradigm shift in the management of advanced ASPS with the introduction of targeted therapies that inhibit angiogenic and growth factor signaling pathways. Tyrosine kinase inhibitors (TKIs) targeting VEGFR and MET, including pazopanib, sunitinib, and cediranib, have demonstrated encouraging results in advanced ASPS, frequently resulting in disease stabilization or partial responses (2, 3). In our patient, both pazopanib and the mTOR inhibitor sirolimus provided temporary stabilization of pulmonary and hepatic metastases but did not prevent progression, highlighting the challenges of acquired resistance and intertumoral heterogeneity (2, 5). Notably, recent evidence suggests that the ASPSCR1-TFE3 fusion upregulates MET signaling, further supporting the rationale for MET inhibitors as promising agents (3). Prospective pediatric trials investigating MET inhibitors in combination with anti-angiogenic therapies are warranted (10). Rare cases of primary pulmonary ASPS in children have also been documented, further illustrating the clinical heterogeneity of this malignancy (11).

Emerging data indicate that ASPS may express immune checkpoint molecules such as PD-L1, providing a rationale for immune checkpoint blockade in refractory or progressive disease (3, 7). While objective responses have been documented in early-phase studies and case series, the overall response rates remain modest, and durable remissions are rare (3, 7). In our patient, pembrolizumab was initiated based on multidisciplinary consensus after the development of multiple new metastatic sites, resulting in disease stabilization without significant immune-related adverse events to date. Further studies are needed to identify predictive biomarkers, refine combination strategies, and determine the optimal timing for integrating immunotherapy into treatment algorithms for pediatric ASPS (3, 7).

An important aspect that warrants discussion is the potential influence of individual patient characteristics, such as age, tumor burden, and metastatic pattern, on treatment response and prognosis. While ASPS tends to exhibit a relatively indolent clinical course compared to other high-grade sarcomas, factors such as younger age, limited metastatic burden at diagnosis, and the feasibility of achieving local control have been associated with better outcomes in retrospective pediatric series (1, 5). In our case, despite aggressive multimodal management, the extensive metastatic disease and repeated progression highlight the prognostic challenges inherent to high-volume, multisite metastatic ASPS in young children.

Effective management of pediatric ASPS requires a coordinated, multidisciplinary approach involving pediatric oncologists, surgical oncologists, pathologists, radiologists, and radiation oncologists (1, 2, 5). Whenever possible, enrollment in clinical trials should be strongly encouraged to facilitate access to novel therapies and contribute to the generation of evidence for this ultra-rare entity (2, 3). Our case underscores the importance of molecular confirmation of diagnosis, careful selection of systemic therapies based on targetable pathways, and the integration of novel treatment strategies.

In conclusion, this case illustrates the paradoxical nature of ASPS as a tumor with slow local growth but aggressive metastatic behavior and intrinsic resistance to standard chemotherapies. While molecularly targeted agents and immunotherapies have provided new hope, their benefits have thus far been partial and transient in many cases. There remains a clear need for further preclinical and clinical research to better understand the molecular drivers of ASPS, optimize treatment sequencing, and explore rational combinations of targeted therapies and immunotherapies. Collaborative international efforts and multicenter pediatric trials are essential to advance therapeutic options and ultimately improve survival outcomes for children affected by this challenging malignancy (1–3, 5, 7).

## Conclusion

Pediatric alveolar soft part sarcoma (ASPS) with metastatic disease is an exceptionally rare and therapeutically challenging sarcoma, often demonstrating intrinsic resistance to conventional cytotoxic chemotherapies. Our case illustrates the necessity of individualized, multimodal management incorporating surgery, radiotherapy, targeted agents, and immune checkpoint inhibition to optimize disease control. While targeted therapies and immunotherapies have shown promise in achieving temporary stabilization, durable responses remain limited, underscoring the importance of continued clinical research. Collaborative international efforts and prospective pediatric trials are urgently needed to refine treatment strategies and improve long-term outcomes for children affected by this aggressive malignancy.

## Data Availability

The original contributions presented in the study are included in the article/Supplementary Material, further inquiries can be directed to the corresponding author.
